# Multimodal Deep Reinforcement Learning with Auxiliary Task for Obstacle Avoidance of Indoor Mobile Robot

**DOI:** 10.3390/s21041363

**Published:** 2021-02-15

**Authors:** Hailuo Song, Ao Li, Tong Wang, Minghui Wang

**Affiliations:** School of Information Science and Technology, University of Science and Technology of China, Hefei 230027, China; shl97@mail.ustc.edu.cn (H.S.); wt18ustc@mail.ustc.edu.cn (T.W.); mhwang@ustc.edu.cn (M.W.)

**Keywords:** obstacle avoidance, mobile robot, multimodal deep reinforcement learning, auxiliary task

## Abstract

It is an essential capability of indoor mobile robots to avoid various kinds of obstacles. Recently, multimodal deep reinforcement learning (DRL) methods have demonstrated great capability for learning control policies in robotics by using different sensors. However, due to the complexity of indoor environment and the heterogeneity of different sensor modalities, it remains an open challenge to obtain reliable and robust multimodal information for obstacle avoidance. In this work, we propose a novel multimodal DRL method with auxiliary task (MDRLAT) for obstacle avoidance of indoor mobile robot. In MDRLAT, a powerful bilinear fusion module is proposed to fully capture the complementary information from two-dimensional (2D) laser range findings and depth images, and the generated multimodal representation is subsequently fed into dueling double deep Q-network to output control commands for mobile robot. In addition, an auxiliary task of velocity estimation is introduced to further improve representation learning in DRL. Experimental results show that MDRLAT achieves remarkable performance in terms of average accumulated reward, convergence speed, and success rate. Moreover, experiments in both virtual and real-world testing environments further demonstrate the outstanding generalization capability of our method.

## 1. Introduction

Avoiding various kinds of obstacles has been regarded as an essential capability of mobile robots and plays an important role in various applications such as autonomous navigation, exploration, and multi-agent coordination. In practice, mobile robots need to perceive objects in their surrounding environment using different sensors (e.g., two-dimensional (2D) laser range finder and cameras) for obstacle avoidance. In the past few years, many works have been proposed to make mobile robots move safely and autonomously in different kinds of unknown environments [1,2]. However, due to the fact that data coming from different sources are typically in different formats and heterogeneous, using multiple sensors to obtain more reliable and robust information for obstacle avoidance remains an open challenge [3].

2D laser range finders have been widely used in mobile robotics for obstacle avoidance [4,5] since it can provide accurate range measurements of surrounding environment in a large horizontal field of view at a fast rate [6]. For example, some studies use a 2D laser range finder to build an occupancy grid map for planning a collision free path [7,8]. Alternatively, Tai et al. propose a deep reinforcement learning (DRL) approach [9], in which sparse laser range findings from raw laser range findings are sampled between −90 and 90 degrees in a fixed angle distribution, and then used as input for a deep neural network to generate velocity control commands for a mobile robot. Furthermore, Xie et al. stack dense 2D laser scans across multiple timestamps and use a 1D convolutional neural network (CNN) to learn efficient features for DRL-based navigation and obstacle avoidance [10].

Despite these 2D laser-only methods showing promising results, the 2D laser range finder may not be sufficient to describe the surrounding environment due to its limited vertical field of view [11], given the fact that the 2D laser range finder cannot detect obstacles lying below or above its line of scanning [12]. In contrast, depth image provides more environment information than the 2D laser range finder does [13], and therefore many researchers attempt to use depth images for obstacle avoidance [13,14,15]. For example, Tai et al. use a 2D CNN to directly process raw depth images for learning efficient features for obstacle avoidance [14]. Similarly, in a DRL-based approach, Wu et al. use stacked depth images and difference images between successive frames as input and utilize a two-stream CNN to process two sets of data, then the extracted features are concatenated and mapped to angular and linear velocities commands for a mobile robot [13]. However, depth image has a limited horizontal field of view that may affect the performance of obstacle avoidance in crowd environment [16].

In the past few years, many studies have been proposed to integrate data from different sensors to obtain more reliable and robust information for obstacle avoidance [17,18,19]. For example, Li et al. propose an approach that generates an integrated local map based on a heuristic fusion method with maps built via 2D laser range findings and depth image synchronously [17]. Similarly, Orlando et al. obtain point clouds from depth image and project them onto the two-dimensional plane, which are further combined with laser data so that the robot could identify obstacles at different heights above the floor [18]. Chavez et al. instead focus on fusing 2D laser range findings and RGB-D images for obstacle avoidance, where each sensor grid map is first constructed, and Bayes rule is then used as a sensor fusion technique to obtain fused occupancy grid map [19]. These studies indicate that 2D laser range findings and depth images are complementary and can be used together to improve the performance of obstacle avoidance.

Recently, there has been an increasing interest in multimodal DRL approaches that integrate multiple sensor modalities for learning control policies and have demonstrated great capability in robotics [20,21]. For example, Qureshi et al. propose a multimodal deep Q-network (DQN) model to learn human-like interactions, in which feature representations from grayscale images and depth images are extracted by a two-stream CNN and then passed through deep neural network to obtain multimodal representation [20]. Similarly, Lee et al. first choose domain specific encoders to capture feature representations from vision and touch and then use a multilayer perceptron to produce multimodal representation for manipulation tasks [21]. To efficiently integrate heterogeneous sensor modalities for autonomous driving, Liu et al. design a sensor dropout method that fuses different features extracted from various sensor modalities using modality-specific feature extraction modules [22]. The successes achieved by these studies indicate the possibility of developing multimodal DRL methods for better control policies in obstacle avoidance. However, due to the complexity of indoor environment, it is still demanding to fully utilize the complementary information between different sensor modalities [23].

In this work, a novel multimodal DRL method with auxiliary task (MDRLAT) is proposed for obstacle avoidance using both 2D laser range findings and depth images. We first use a two-stream CNN to extract feature representations from different sensor modalities. To effectively fuse the extracted feature representations, a bilinear fusion (BF) module is designed to generate multimodal representation by fully capturing the complementary information across different sensor modalities, which is subsequently mapped to control commands by dueling double deep Q-network (D3QN). In addition, for improving representation learning in DRL, we introduce an auxiliary task of velocity estimation that requires the robot to estimate its velocities from the generated multimodal representation. Specifically, this auxiliary task shares the neural network that the robot uses to act and therefore introduces extra gradient during back-propagation. By using the jointly learned multimodal representation, the robot learns to optimize reward faster and achieves better policies at the end of training. To assess the performance of our method, several different virtual environments were used for training and testing and the experimental results show that MDRLAT achieves remarkable improvements over baseline methods in terms of average accumulated reward, convergence speed, and success rate. Moreover, experiments implemented in various real-world scenarios further demonstrate the outstanding generalization capability of our proposed method. The code of MDRLAT is available on Github (https://github.com/hailuoS/MDRLAT (accessed on 6 February 2021)).

## 2. Materials and Methods

### 2.1. Problem Definition

We consider the obstacle avoidance problem as a Markov Decision Process (MDP) and use a tuple $M=\left(S,A,P,R,\gamma \right)$ to define it, in which $S,A,P,R,\gamma \in \left[0,1\right]$ are state space, action space, transition function (describing the probability of transiting to next state), reward function, and discount factor (allowing to control the influence of future rewards), respectively [11,24]. Specifically, at time step t, robot chooses an action $a_{t}\in A$ based on current input state $s_{t}\in S$, transits to next state $s_{t+1}$ according to transition function P, and obtains an immediate reward $r_{t}=R\left(s_{t},a_{t}\right)$ from environment according to reward function R. In an MDP, a policy π(a|s) is used to map state s to an action a and we can assess the superiority of a policy π by using an action-value function (*Q*-value) that is formulated as below:

(1) $$Q^{\pi }\left(s,a\right)=E^{\pi }[\overset{+\infty }{\underset{t=0}{\sum }}\gamma^{t}R\left(s_{t},a_{t}\right)|s_{0}=s,a_{0}=a]$$

Q-value is the expectation of discounted cumulative rewards when the robot first chooses an action a at state s and act according to policy π in subsequent time steps [24]. The goal of MDP is finding a policy to maximize the expected discounted cumulative reward, and Q-learning algorithms are often be utilized to deal with this problem through the following Bellman equation to approximate the optimal Q-value iteratively:

(2) $$Q^{*}\left(s_{t},a_{t}\right)=R\left(s_{t},a_{t}\right)+\gamma \underset{a_{t+1}}{\max}Q\left(s_{t+1},a_{t+1}\right)$$

Then, an optimal policy can be derived from the optimal *Q*-value by selecting the action that has the highest value in each state [25].

In this work, the state $s_{t}$ includes the 2D laser range findings $l_{t}$ and depth images $d_{t}$. Specifically, $l_{t}$ consists of the measurements of four consecutive frames from a 270-degree 2D laser range finder that produces 512 distance values per scanning (i.e., $l_{t}\in {\mathbb{R}}^{4\times 512}$), and $d_{t}$ includes depth images from four consecutive frames obtained by depth camera that are all resized to 128 × 160 (i.e., $d_{t}\in {\mathbb{R}}^{4\times 128\times 160}$). Moreover, as shown in Table 1, the discrete action space contains a set of control commands, each including both linear and angular velocity (i.e., $a_{t}=\left[v_{t},\omega_{t}\right]$). Apart from state space and action space, a reasonable reward function is indispensable in reinforcement learning and we carefully design it as below:

(3) $$r_{t}={\left(r^{c}\right)}_{t}+{\left(r^{d}\right)}_{t}+{\left(r^{\omega }\right)}_{t}$$

Inspired by Wu et al. [11], ${\left(r^{c}\right)}_{t}$ is given as:

(4) $${\left(r^{c}\right)}_{t}=\left\{\begin{array}{c} \;-10\;\;\;\;\;\;\;\;\;\;\;\;\;\;\;\;\;\;\;\;\;\;\;\;\;if\;d_{min}<0.2\\ \lambda_{c}v_{t}^{2}\cos(v_{t}\omega_{t})\;\;\;\;\;\;\;\;otherwise \end{array}\right.$$

where $d_{min}$ is the minimum value of 2D laser range findings. In general, the robot will get a large penalty if a collision occurs, otherwise it is expected to move forward rapidly in the environment and only turn when it is necessary to avoid obstacles. In addition, a penalty for moving too close to an obstacle is given by:

(5) $${\left(r^{d}\right)}_{t}=\lambda_{d}\left(0.4/d_{min}\right)\;\;if\;\;0.2<d_{min}<0.4$$

Moreover, in order to make robot move smoothly, a small penalty to mitigate the left-right swing behavior is given as:

(6) $${\left(r^{\omega }\right)}_{t}=\lambda_{\omega }\left(\left|\omega_{t}-\omega_{t-1}\right|\right)\;\;if\;\;\omega_{t}*\omega_{t-1}<0$$

In above equations, the constants $\lambda_{c}$, $\lambda_{d}$, and $\lambda_{\omega }$ are weighting factors and we set $\lambda_{c}=2$, $\lambda_{d}=-0.05$, and $\lambda_{\omega }=-0.05$ respectively in the training procedure.

### 2.2. Network Architecture

**Figure 1:**
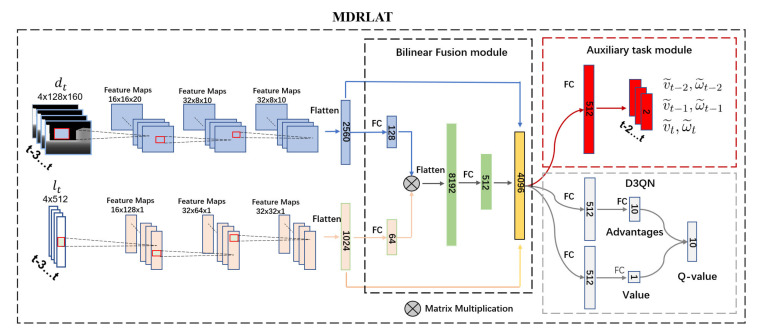
Network architecture of the proposed MDRLAT.

In this section, we describe the network architecture of our proposed MDRLAT, which consists of a two-stream CNN and a BF module to generate multimodal representation from different sensor modalities, a D3QN to obtain control commands from the multimodal representation, and an auxiliary task module for improving representation learning in DRL. The overall network architecture is shown in Figure 1 and following subsections describe each part of MDRLAT in detail.

#### 2.2.1. Network Architecture for Multimodal Representation

The proposed MDRLAT adopts a two-stream CNN to extract feature representations from different sensor modalities and a BF module is proposed to effectively fuse the extracted feature representations and generate multimodal representation. Specifically, the two-stream CNN consists of multiple 2D and 1D convolutional layers to process $d_{t}$ and $l_{t}$, respectively. For one stream of CNNs, the first convolutional layer converts $d_{t}$ to feature maps through 16 filters with kernel size 10 × 14 and stride 8. Then, the second convolutional layer processes the feature maps through 32 filters with kernel size 4 × 4 and stride 2 and the final convolutional layer subsequently produces 32 8 × 10 feature maps by using filters with kernel size 3 × 3 and stride 1. For the other stream of CNNs, the first convolutional layer processes $l_{t}$ via 16 filters with kernel size 5 and stride 4. The second convolutional layer contains 32 filters with a kernel size of 3 and stride 2, and the third convolutional layer generates 32 32 × 1 feature maps using 32 filters with kernel size 3 and stride 2. In practice, all the convolutional layers are followed by the rectified linear unit (ReLU) activation function [26]. Finally, the output from each stream is flattened and regarded as the feature representation of the corresponding sensor modality.

In order to effectively fuse the feature representations extracted by the two-stream CNN, we design a BF module to fully capture the complementary information across different sensor modalities. Firstly, by using fully connected layers, the extracted features of 2D laser range findings and depth images are embedded into feature vectors denoted as $l\in {\mathbb{R}}^{L_{l}\times 1}$ and $d\in {\mathbb{R}}^{L_{d}\times 1}$, respectively. Then, we adopt a bilinear function to fuse l and d, which can be formulated as:

(7) $$f_{k}^{b}=\mathrm{ReLU}\left(W_{k}\mathrm{vec}\left(dl^{T}\right)+b_{k}\right)$$

where $f_{k}^{b}$ is the k-th value of $f^{b}\in {\mathbb{R}}^{K\times 1}$, $\mathrm{vec}\left(\cdot \right)$ is the operation to unfold a matrix to a vector, $W_{k}$ and $b_{k}$ are learnable weight matrix and bias term, respectively. Specifically, Equation (7) can be written by the general form of bilinear model [27]:

(8) $$f_{k}^{b}=\mathrm{ReLU}(\overset{L_{d}}{\underset{i=1}{\sum }}\overset{L_{l}}{\underset{j=1}{\sum }}w_{ijk}d_{i}l_{j}+b_{k})$$

where $w_{ijk}$ is the entry of $W_{k}$, and $d_{i}$ and $l_{j}$ are the i-th, j-th value of d and l, respectively. In this work, $L_{d}$, $L_{l}$, and K are set to 128, 64, and 512, respectively. Finally, $f^{b}$ is concatenated with feature representations of 2D laser range findings and depth images to obtain multimodal representation as the input of D3QN.

#### 2.2.2. Dueling Double Deep Q-Network

In order to generate control commands for the mobile robot from the obtained multimodal representation, we select the widely used D3QN that is an extension of DQN by leveraging the techniques of double Q-learning [25] and dueling architecture [28]. Specifically, DQN includes two deep neural networks with same architecture, one is an online network with parameter θ for selecting action and the other is a target network with parameter $\theta^{-}$ for generating target Q-value $y_{t}$:

(9) $$y_{t}=r_{t}+\gamma \underset{a_{t+1}}{\max}Q\left(s_{t+1,}a_{t+1};\theta^{-}\right)$$

It is noteworthy that the maximum operation in Equation (9) leads to overestimation of *Q*-values. In order to alleviate this problem, Van et al. [25] propose double DQN that decouples action selection from action evaluation and $y_{t}$ can be rewritten as:

(10) $$y_{t}=r_{t}+\gamma Q(s_{t+1},\underset{a_{t+1}}{\mathrm{argmax}}Q\left(s_{t+1},a_{t+1};\theta \right);\theta^{-})$$

To further promote the performance of double DQN, D3QN uses a dueling architecture that decomposes Q-value into state value and action advantages. As shown in Figure 1, the state value function and action advantage function are separately estimated by using a dueling architecture with two streams of fully connected layers. Then, the *Q*-value of each action is generated by combining the obtained state value and action advantages, which can be formulated as:

(11) $$Q\left(s,a\right)=V\left(s\right)+(A\left(s,a\right)-\frac{1}{\left|A\right|}\underset{a^{\prime }}{\sum }A\left(s,a\right))$$

where $V\left(s\right)$ represents state value function, $A\left(s,a\right)$ is action advantage function, and $\left|A\right|$ is the size of action space.

#### 2.2.3. Auxiliary Task Module

A common problem in DRL is sample inefficiency that means the agent (i.e., robot) often requires a prohibitively large number of samples to learn a policy. Recently, some researchers showed that augmenting the RL agent with auxiliary tasks can improve representation learning and alleviate the sample inefficiency problem [29,30]. Inspired by these works, we introduced an auxiliary task of velocity estimation that requires the robot to estimate its real-valued velocities from the generated multimodal representation. Specifically, we define ${\bar{v}}_{t}$ and ${\bar{\omega }}_{t}$ as the ground truth linear and angular velocity of robot obtained from training environment at time step t, respectively. In practice, since the input state $s_{t}$ includes four consecutive frames from time step t−3 to t, we leverage a neural network to estimate the velocities at times t−2, t−1, and t from the generated multimodal representation, as illustrated in Figure 1. Accordingly, we formulate the loss function of the auxiliary task, $L_{AT}$, by using mean squared error between the estimated velocities and ground truth as follows:

(12) $$L_{AT}=\overset{t}{\underset{i=t-2}{\sum }}\left({\left({\bar{v}}_{i}-{\tilde{v}}_{i}\right)}^{2}+{\left({\bar{\omega }}_{i}-{\tilde{\omega }}_{i}\right)}^{2}\right)$$

where $\tilde{v}$ and $\tilde{\omega }$ are the estimated linear and angular velocity, respectively. During the training process, this auxiliary task module shares the multimodal deep neural network with D3QN and therefore introduces extra gradient for improving representation learning in DRL.

### 2.3. Training Framework

**Figure 2:**
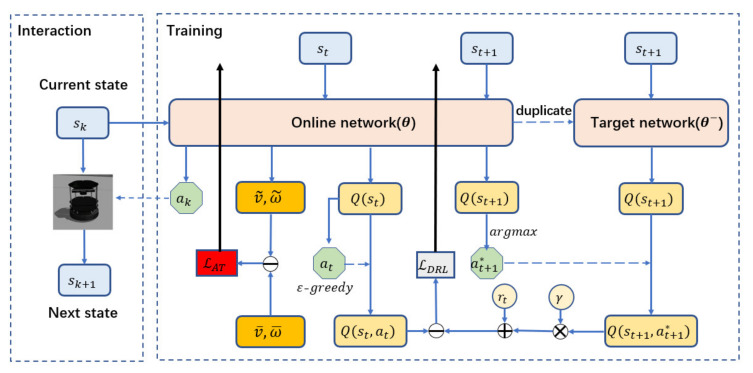
Training framework of the proposed MDRLAT.

The training framework of MDRLAT is shown in Figure 2 and the pseudo-code is described in Algorithm 1. The online network and the target network are used to select action and generate target Q-value, respectively. At each time step, the mobile robot implements the control command determined by online network based on current state, transits to next state, and gets a reward from the environment. In the meantime, we store this interaction in an experience replay buffer that is updated continuously with a maximum capacity of 20,000.

In the beginning of training, we initialize the parameters of online network randomly and duplicate them to the target network. After that, at each training step, the parameters of online network are updated by using a batch of interactions that are sampled randomly from experience replay buffer. Specifically, online network outputs a vector $Q\left(s_{t};\theta \right)$ that contains Q-values of each action based on current input state $s_{t}$. Then, the action $a_{t}$ to be executed is determined by ε-greedy strategy [31] and its Q-value $Q\left(s_{t},a_{t};\theta \right)$ is also obtained accordingly. At the same time, the next state $s_{t+1}$ is passed through the online network to determine optimal action $a_{t+1}$ and also fed into the target network to compute target Q-value $y_{t}$ of the determined action $a_{t}$ by using Equation (10). Based on the *Q*-values obtained by online network and target network, we define the loss function of DRL as below:

(13) $$L_{DRL}={\left(y_{t}-Q\left(s_{t},a_{t};\theta \right)\right)}^{2}$$

In addition, the online network is also used for the proposed auxiliary task by using Equation (12). Therefore, the parameters of the multimodal representation in the online network are updated simultaneously by two-part back-propagation error and two Adam optimizers [32] are used with a same learning rate of 0.0001. On the other hand, the target network is not trainable, and we therefore synchronize its parameters with those of the online network every 1000 steps.

**Algorithm 1**: MDRLAT1: Initialize experience replay buffer D to capacity N, parameters of online network θ, parameters of target network $\theta^{-}=\theta$, frequency $N_{t}$ to update target network2: **for**
t=1, 2,…, T
**do**3:   Obtain $s_{k}=\left[l_{k},d_{k}\right]$ from the environment, with probability ε select a random action $a_{k}$, otherwise select $a_{k}=\underset{a}{\mathrm{argmax}}Q\left(s_{k},a;\theta \right)$4:   Execute action $a_{k}$, transit to next state $s_{k+1}$ and receive a reward $r_{k}$5:   Store transition $\left(s_{k},a_{k},r_{k},s_{k+1}\right)$ in D6:   Randomly sample a batch of $N_{B}$ transition $\left(s_{t},a_{t},r_{t},s_{t+1}\right)$ from D7:   **if** episode terminates at step t+1
**then**8:    Set $y_{t}=r_{t}$9:   **else**10:    Set $y_{t}=r_{t}+\gamma Q(s_{t+1},\underset{a_{t+1}}{\mathrm{argmax}}Q\left(s_{t+1},a_{t+1};\theta \right);\theta^{-})$11:   **end if**12:   Compute loss $L_{DRL}={\left(y_{t}-Q\left(s_{t},a_{t};\theta \right)\right)}^{2}$13:   Feed $s_{t}$ into the online network to get estimated velocity $\tilde{v}$, $\tilde{\omega }$
14:   Compute loss $L_{AT}=\overset{t}{\underset{i=t-2}{\sum }}\left({\left({\bar{v}}_{i}-{\tilde{v}}_{i}\right)}^{2}+{\left({\bar{\omega }}_{i}-{\tilde{\omega }}_{i}\right)}^{2}\right)$15:   Update the parameters of the online network θ16:   **if**
t mod $N_{t}=0$
**then**17:    Update the parameters of the target network $\theta^{-}\leftarrow \theta$18:   **end if**19: **end for**

## 3. Results

### 3.1. Experiments in Virtual Environments

**Figure 3:**
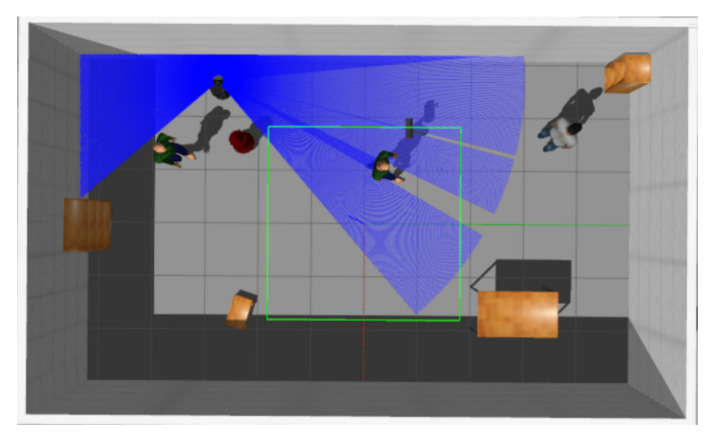
The virtual training environment.

In this section, we evaluate the performance of MDRLAT in a variety of virtual environments. As shown in Figure 3, we created a 6 m × 10 m virtual training environment containing different static and dynamic obstacles in Gazebo [33] and used a simulated turtlebot2 robot to interact with environment. The proposed method was implemented with Tensorflow [34] and trained on a computer with Intel(R) Xeon(R) E5-2630 2.30GHz CPU, 64GB RAM, and NVIDIA GeForce GTX 1080 Ti GPU. During training and testing, the 2D laser range findings and depth images were captured by a simulated Hokuyo 2D laser range finder and Kinect, respectively, and the output control commands are transmitted to mobile robot via robot operating system (ROS) messages. In addition, we calculate reward function and the loss function of auxiliary task by using the subscribed odometry message in training process.

In order to illustrate the effectiveness of the proposed method, we compared MDRLAT with a series of baseline methods: (1) Laser-only: in this case, we directly feed the feature representation extracted from 2D laser range findings into D3QN, (2) Depth-only: in this case, we directly feed the feature representation extracted from depth images into D3QN, (3) Multi: in this case, we directly concatenate the feature representations extracted from different sensor modalities, (4) Multi-AT: in this case, we adopt the auxiliary task module in the multi method, (5) Multi-BF: in this case, we adopt the BF module in the multi method. All methods were trained for 4e5 steps from scratch and we set batch size and discount factor to 32 and 0.99, respectively. In addition, ε-greedy strategy was used to determine action and ε was initialized to 0.1 and decayed linearly to 0.0001 within the first 20,000 training steps. In addition, we set the maximum steps of an episode to 500, which means an episode is terminated if no collision occurs after 500 steps.

**Figure 4:**
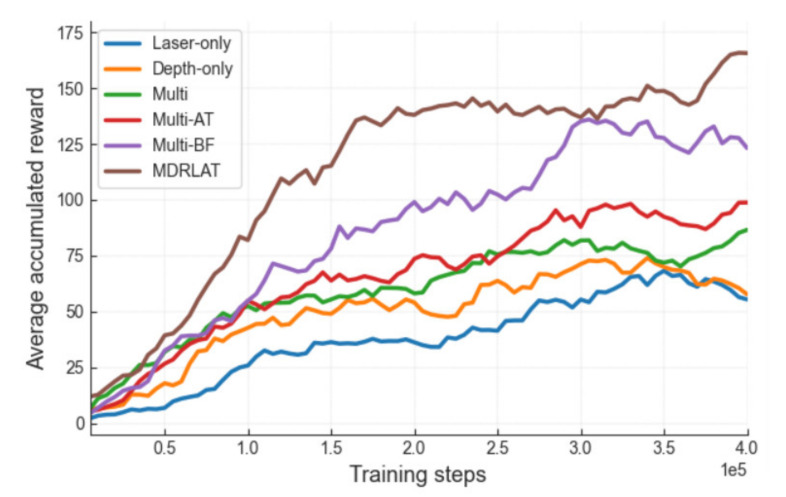
Smoothed training curves of average accumulated rewards obtained by each method at different training steps. Laser-only, directly feed the feature representation extracted from two-dimensional (2D) laser range findings into dueling double deep Q-network (D3QN); depth-only, directly feed the feature representation extracted from depth images into D3QN; multi, directly concatenate the feature representations extracted from different sensor modalities; multi-AT, adopt the auxiliary task module in the multi method; multi-BF, adopt bilinear fusion (BF) module in the multi method; MDRLAT, our proposed method.

During the training process, we first calculated the accumulation of immediate rewards after every 5000 training steps, and the accumulated reward averaged by five episodes is used to evaluate each method. The smoothed training curves of average accumulated rewards obtained by all methods are shown in Figure 4. We can see that the laser-only method obtained the lowest average accumulated reward during the training while the depth-only method achieved better performance. One possible reason for this phenomenon is that the 2D laser range finder cannot detect the obstacle lying below its scanning field [12]. Compared to these methods, the multi method leads to a higher average accumulated reward by using multiple sensors that can obtain more information of surrounding environment. More importantly, the multi-BF method outperformed the multi method with a remarkable improvement on average accumulated reward throughout the training, indicating that the BF module can further improve the performance of obstacle avoidance by fully capturing the complementary information across different sensor modalities. Moreover, the introduction of the auxiliary task module boosted the performance of both multi method and multi-BF method, making MDRLAT converge much faster and achieve higher average accumulated reward throughout the training. Taken together, the proposed MDRLAT exhibits the most prominent performance during the training process among all the methods investigated in this work.

**Figure 5:**
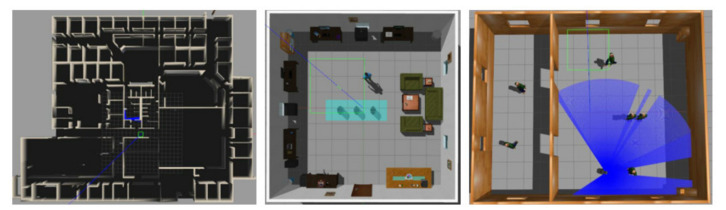
The virtual testing environments used in this work.

Apart from average accumulated reward, we also compare the success rate of each method in this work. By following previous work [13], an episode is considered to be successful if no collision occurs within 300 steps and the success rate represents the ratio of successful episodes in 50 episodes. In order to assess the generalization capability, we tested all methods in three different kinds of virtual environments directly without any fine-tuning. As shown in Figure 5, the first one is an office environment containing many corridors and narrow rooms. The second one is a more realistic environment with a lot of unseen furniture created by [35] and the last one is a dynamic environment with a number of pedestrians. In short, these testing environments are more different and complicated compared to the training environment.

We tested all methods five times in each environment and the average and standard deviation of success rate are shown in Table 2. It can be observed that all methods using multiple sensors produced higher performance than the laser-only method and depth-only method, which indicates that 2D laser range findings and depth images are complementary and can be used together to improve the performance of obstacle avoidance. Furthermore, we find that the multi-BF method consistently performs better than multi method. For example, the average success rates of multi-BF method were 89.6% and 79.6% in the first and third environments respectively, which have 12.8% and 8.8% improvement over multi method, respectively. These results suggest the great strength of the BF module in capturing the complementary information across different sensor modalities for obstacle avoidance. Meanwhile, we also find the multi-AT method can obtain higher average success rate than the multi method. For example, the average success rates achieved by the multi-AT method reach 86.0% and 78.8% in the first and second environment, respectively, compared with 76.8% and 73.6% obtained by the multi method, which demonstrates the outstanding effectiveness of auxiliary task module for improving representation learning in DRL. Moreover, by integrating both the BF module and the auxiliary task module, MDRLAT achieves the best performance across all three environments with remarkable improvements on average success rate. Take the first environment as an example, MDRLAT manages to obtain an average success rate of 94.4%, which achieves an improvement of 17.6%, 8.4%, and 4.8% over the multi method, multi-AT method, and multi-BF method, respectively. In conclusion, compared with other methods, the proposed MDRLAT exhibits superior generalization capability of obstacle avoidance.

**Figure 6:**
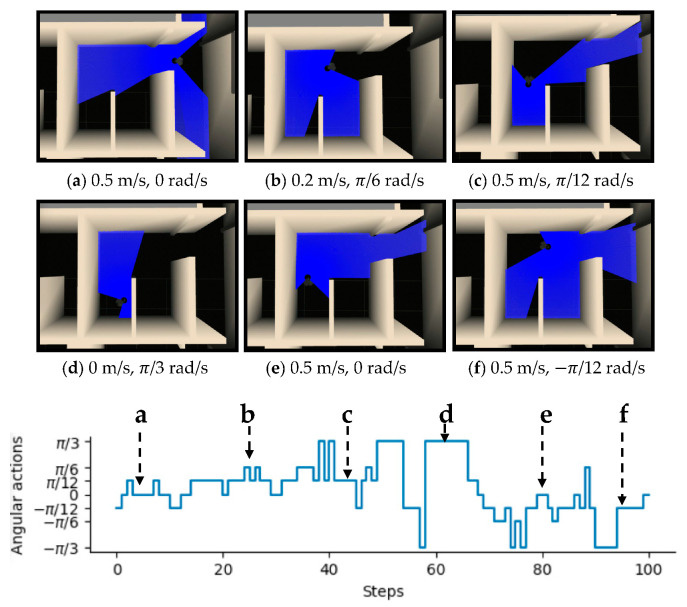
An example of dealing with dead end in virtual testing environment. The subfigures show the top view of six intermediate time steps and the sub-captions indicate the corresponding linear and angular velocity commands. Angular velocity commands in all time steps are provided at the bottom.

**Figure 7:**
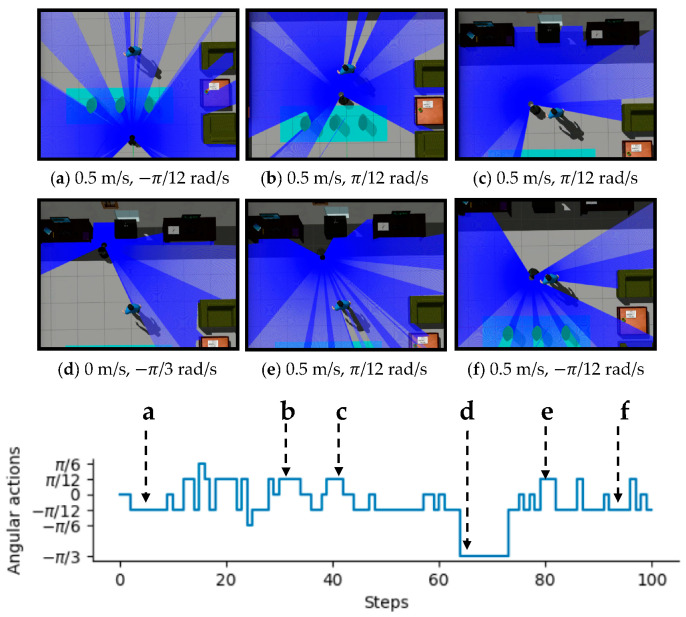
An example of dealing with different kinds of obstacles in virtual testing environment. The subfigures show the top view of six intermediate time steps and the sub-captions indicate the corresponding linear and angular velocity commands. Angular velocity commands in all time steps are provided at the bottom.

To intuitively illustrate the performance of the proposed method, two examples of control commands produced by MDRLAT in different scenarios are shown in Figure 6 and Figure 7. For the convenience of description, the top view of six intermediate time steps is presented and the sub-caption indicates the corresponding linear and angular velocity commands in each example. Meanwhile, angular velocity commands in all time steps are provided at the bottom. As shown in Figure 6, in the beginning, the robot executes a large linear velocity command to pass through the door. Subsequently, the robot turns left to prevent from bumping into the wall and enters the narrow room. Later on, a zero linear velocity and a large angular velocity are derived so that robot can drive itself out of a dead end without any collision. Another example is shown in Figure 7, after passing through the table, the robot is controlled to make a left turn to avoid collision with the person. Subsequently, the robot moves forward rapidly and then changes its orientation to avoid the desk and office box. Taken together, these examples demonstrate the ability of our proposed method to deal with dead ends and different kinds of obstacles in virtual testing environments.

**Figure 8:**
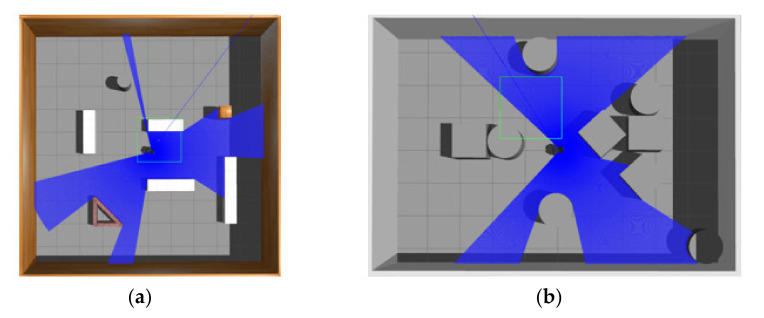
The training and testing environments for navigation task. (**a**) training environment (**b**) testing environment.

**Figure 9:**
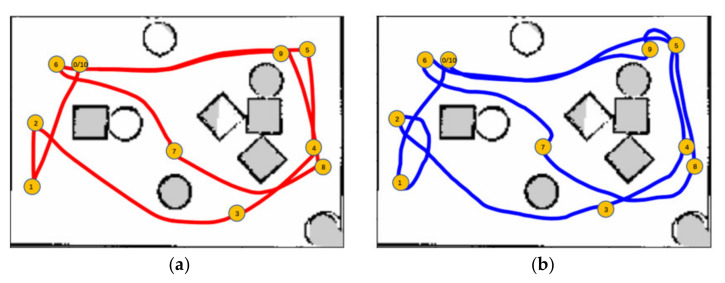
The trajectories generated by Move_Base and MDRLAT. (**a**) Move_Base (**b**) MDRLAT.

Although we focus on obstacle avoidance task in this work, the proposed MDRLAT can be extended for navigation task by adding goal information (e.g., the coordinate of goal in the robot’s local coordinate frame). Here, we conducted a simple experiment by concatenating the goal information and the multimodal representation and using the reward function designed by Xie et al. [10]. The training and testing environments for MDRLAT are shown in Figure 8. During testing, a widely used motion planner Move_Base [36] was used for comparison and the global map of testing environment was built for Move_Base to calculate the path. The generated trajectories of both methods are illustrated in Figure 9. We found that although the trajectory generated by MDRLAT was more tortuous than Move_Base, the robot could safely reach all goals without a global map in a previous unseen environment, which demonstrates the potential of MDRLAT for navigation task.

### 3.2. Experiments in Real-World Environments

**Figure 10:**
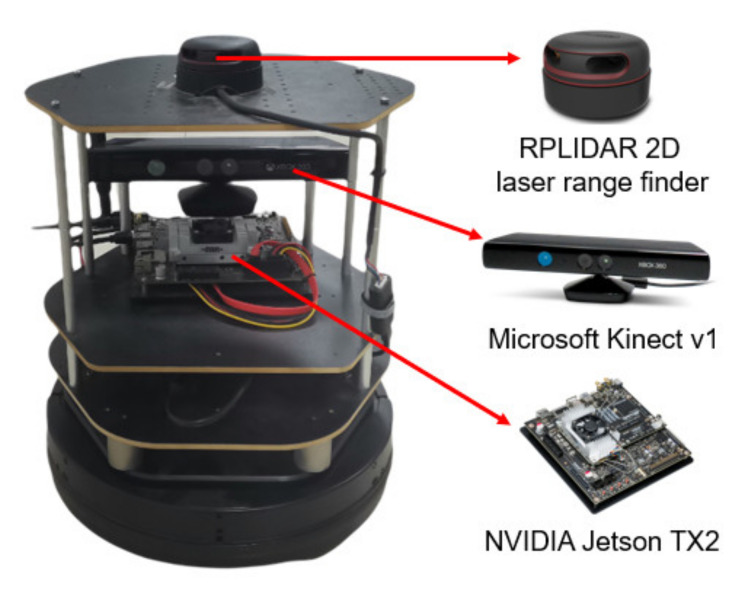
A turtlebot2 used for experiments in real-world environments.

**Figure 11:**
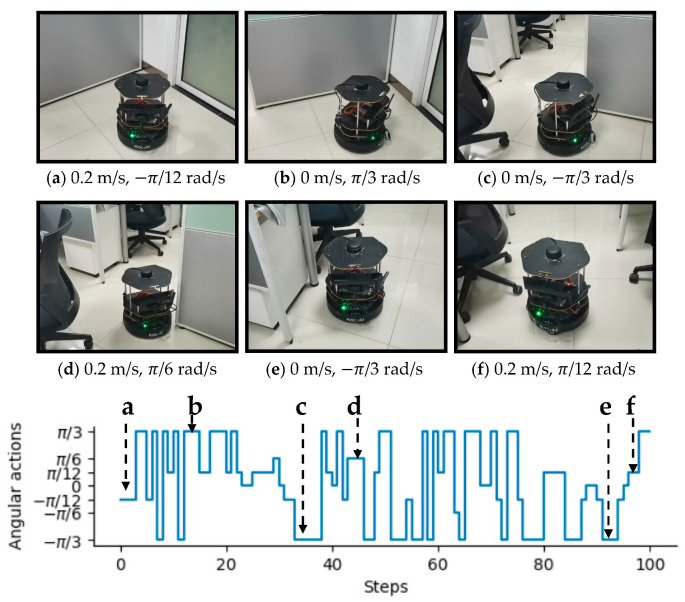
An example of dealing with obstacles in a real-world office environment. The subfigures show the third-person view of six intermediate time steps and the sub-captions indicate the corresponding linear and angular velocity commands. Angular velocity commands in all time steps are provided at the bottom.

In this section, a variety of experiments were implemented in real-world scenarios to further assess the performance of the proposed MDRLAT. As shown in Figure 10, we used an NVIDIA Jetson TX2 as computing platform and an open source hardware platform turtlebot2 [37] equipped with a RPLIDAR 2D laser range finder and a Microsoft Kinect v1 camera for experiments. The control commands determined by MDRLAT were transmitted to the robot through ROS messages. Firstly, we tested our proposed method in an office environment, the third-person view of intermediate time steps is presented in Figure 11 and the sub-caption indicates the corresponding linear and angular velocity commands. In the beginning, the robot was running into a dead end and then it changed direction to the left to prevent from bumping into the office cubicle. Subsequently, when a previously unseen obstacle (office chair) was detected, a large angular velocity was selected for the robot to turn right to avoid collision. Then, the robot executed a small angular velocity to adjust its orientation so that it can pass through between office chair and office cubicle without any collision. After that, the robot moved forward with a small linear velocity in the constrained space and then made a right turn with a large angular velocity to avoid another office chair. This suggests that by following the control commands determined by MDRLAT, the robot can move safely in cluttered office environment with objects absent in the training environment.

**Figure 12:**
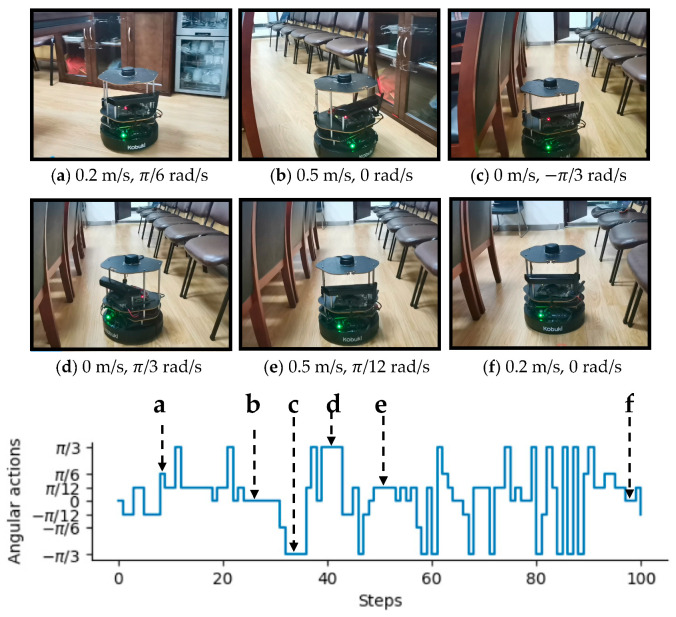
An example of passing through narrow space in a real-world meeting room between two rows of chairs. The subfigures show the third-person view of six intermediate time steps and the sub-captions indicate the corresponding linear and angular velocity commands. Angular velocity commands in all time steps are provided at the bottom.

**Figure 13:**
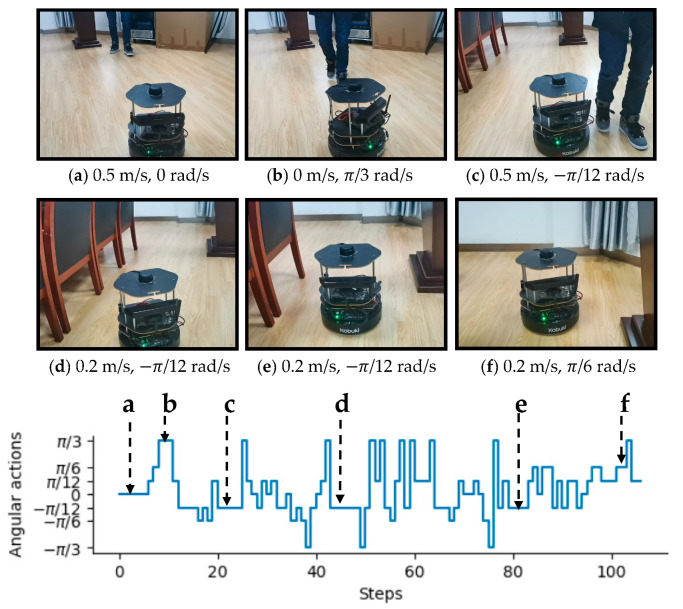
An example of dealing with dynamic obstacle in a real-world meeting room. The subfigures show the third-person view of six intermediate time steps and the sub-captions indicate the corresponding linear and angular velocity commands. Angular velocity commands in all time steps are provided at the bottom.

In addition, our MDRLAT was evaluated in a meeting room as well. As depicted in Figure 12, in the beginning, the robot executed a small angular velocity and turned left to prevent from bumping into the cabinet. Subsequently, the robot travelled forward with maximum linear velocity until a right turn was needed to avoid colliding with chairs on the left. Later on, a left turn command was derived to adjust the orientation of the robot so that it could move safely in the narrow space between two rows of chairs. Finally, an example of dealing with dynamic obstacle is illustrated in Figure 13. It can be observed that the robot moved forward with the maximum linear velocity and a zero angular velocity when the pedestrian was far away. However, as the pedestrian kept moving ahead, the robot received a large angular velocity command and turns left rapidly to avoid the pedestrian. After that, the robot continued to travel forward with the maximum linear velocity and then turned right to avoid chairs. Later on, a decreased linear velocity was derived to control the robot to pass through between chairs and lecture table safely. Taken together, these experiments validate the outstanding performance of the proposed MDRLAT, which can enable a mobile robot to avoid various obstacles in different complicated real-world environments.

## 4. Conclusions

In this work, we propose a novel multimodal DRL method, MDRLAT, for obstacle avoidance of indoor mobile robot. Specifically, in MDRLAT a powerful BF module is proposed to fully capture the complementary information from 2D laser range findings and depth images, and the generated multimodal representation is subsequently fed into D3QN to output control commands for mobile robot. In addition, an auxiliary task of velocity estimation is introduced to further improve representation learning in DRL. We carefully assess the performance of the proposed method in various virtual environments and the experimental results show the remarkable performance of MDRLAT in terms of average accumulated reward, convergence speed, and success rate. Moreover, experiments in both virtual and real-world testing environments further demonstrate the outstanding generalization capability of our method. Despite the promising results achieved by MDRLAT, there is still room for further improvements. Firstly, some other information (e.g., optical flow) is also useful for obstacle avoidance task [38], which could be combined with 2D laser range findings and depth images to further enhance the performance. Secondly, although we use multiple sensors to obtain information of surrounding environment, the input information may still be insufficient since only four consecutive frames of 2D laser range findings and depth images are fed into deep neural network, which could be alleviated by taking more past memories into consideration. Finally, we will extend this work to deal with more complicated tasks like multi-robot obstacle avoidance by using multi-agent DRL [39].

## Figures and Tables

**Table 1:** Action space defined in this work.

Action	0	1	2	3	4	5	6	7	8	9
$v\left(m/s\right)$	0.5	0.5	0.5	0.2	0.2	0.2	0.2	0.2	0	0
$\omega \left(rad/s\right)$	−π/12	0	π/12	−π/6	−π/12	0	π/12	π/6	−π/3	π/3

**Table 2:** Average success rate (%) and standard deviation obtained by different methods in three testing environments.

Env	Laser-Only	Depth-Only	Multi	Multi-AT	Multi-BF	MDRLAT
**Env1**	67.6 ± 4.8	69.2 ± 3.5	76.8 ± 4.1	86.0 ± 4.2	89.6 ± 1.5	94.4 ± 2.3
**Env2**	61.6 ± 4.6	55.2 ± 4.8	73.6 ± 5.3	78.8 ± 2.7	81.2 ± 3.0	87.2 ± 2.4
**Env3**	64.8 ± 3.2	63.2 ± 2.7	70.8 ± 4.1	78.4 ± 5.0	79.6 ± 3.4	83.6 ± 3.2

Laser-only, directly feed the feature representation extracted from 2D laser range findings into D3QN; depth-only, directly feed the feature representation extracted from depth images into D3QN; multi, directly concatenate the feature representations extracted from different sensor modalities; multi-AT, adopt the auxiliary task module in the multi method; multi-BF, adopt BF module in the multi method; MDRLAT, our proposed method.

## Data Availability

Not applicable.
